# Biological Disease-Modifying Antirheumatic Drugs Decrease Uric Acid Levels in the Sera of Patients with Psoriatic Arthritis

**DOI:** 10.3390/cimb47030142

**Published:** 2025-02-22

**Authors:** Dijana Perković, Marin Petrić, Maja Maleš, Ivana Erceg Maleš, Mislav Radić

**Affiliations:** 1 Division of Clinical Immunology and Rheumatology, Department of Internal Medicine, University Hospital of Split, 21000 Split, Croatia; dijana.perkovic@hotmail.com (D.P.); ivana_erceg2206@yahoo.com (I.E.M.); mislavradic@gmail.com (M.R.); 2 Department of Internal Medicine, School of Medicine, University of Split, 21000 Split, Croatia; maja.males13@gmail.com

**Keywords:** biological disease-modifying antirheumatic drugs, interleukin-17, psoriatic arthritis, tumor necrosis factor alpha, uric acid

## Abstract

Objectives: There are many explanations for increased levels of serum uric acid (SUA) in patients with psoriatic arthritis (PsA), but correlation with different treatment options in PsA is not well elucidated. Our aim was to determine the effects of biological disease-modifying antirheumatic drugs (bDMARDs) on SUA levels in patients with PsA. Materials and methods: We analyzed the data of PsA patients treated with different bDMARDs from January 2007 to June 2021. Patients treated with interleukin-17 (IL-17) inhibitors (secukinumab and ixekizumab) and tumor necrosis factor α (TNFα) inhibitors (golimumab, infliximab, adalimumab, certolizumab pegol, and etanercept) were included. Results: A total of 87 patients were included. The SUA levels decreased in 60 (69%) patients after a 3–6-month-long follow-up, and in 25 (28.7%), we noticed an increase. The average decrease in SUA levels was 9.4 ± 49.5 µmol/L (*p* = 0.039); for TNFα patients, it was 7.3 ± 59.8 µmol/L (*p* = 0.386), and for IL-17 patients, it was 12.6 ± 28.4 µmol/L (*p* = 0.013). The levels of SUA decreased in 81.8% of patients treated with infliximab, as well as in 76% of those treated with secukinumab and in 72.7% of those treated with etanercept. The largest average decrease in SUA levels was recorded in the group treated with golimumab (23 µmol/L). Conclusions: A significant decrease in SUA levels was noticed, especially in patients treated with IL-17 inhibitors. Further studies should identify which bDMARD is the most potent in the lowering of SUA levels. bDMARDs were efficient in PsA disease activity.

## 1. Introduction

Psoriatic arthritis (PsA) is a heterogeneous chronic inflammatory disease characterized by peripheral and axial arthritis, enthesitis, and psoriasis (PsO). The disease is burdened with comorbidities such as hypertension, diabetes mellitus, metabolic syndrome, and cardiovascular (CV) comorbidities [1,2]. The incidence of hyperuricemia is almost three times higher in patients with PsA than in the general population [3,4]. Proinflammatory cytokines responsible for a systemic inflammatory process in PsA are related to increased levels of SUA [5,6]. Keratinocyte hyperproliferation, increased cell apoptosis, and necrosis due to inflammation are related to increases in SUA levels in PsO or PsA. An additional factor is reduced renal and extra-renal SUA clearance [7]. Increased levels of serum uric acid (SUA) are also recognized as an independent risk factor for future major CV events [8,9]. Hyperuricemia is defined as SUA levels that are ≥450 µmol/L (7.6 mg/dL) for males and ≥360 µmol/L (6 mg/dL) for females [3,10].

Biological disease-modifying antirheumatic drugs (bDMARDs) are different monoclonal antibodies used for the treatment of PsA when conventional synthetic disease-modifying antirheumatic drugs (csDMARDs) are not effective. The most commonly used bDMARDs are tumor necrosis factor α (TNFα) inhibitors, interleukin-17 (IL-17) inhibitors, IL-12/23 inhibitors, and IL-23 inhibitors. These drugs have significantly changed the course of PsA [11].

The aim of our study was to determine the effects of bDMARD therapy on SUA levels in patients with PsA.

## 2. Patients and Methods

**Figure 1:**
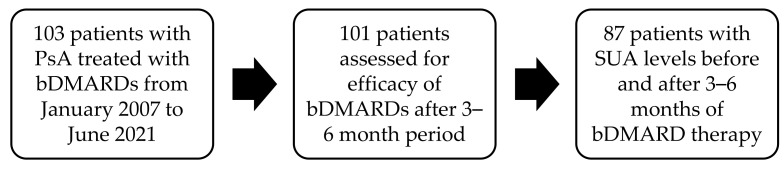
Flowchart of included patients’ data. bDMARD—biological disease-modifying antirheumatic drug, PsA—psoriatic arthritis, SUA—serum uric acid.

This retrospective single-center study included adult PsA patients (≥18 years) treated with bDMARDs in the Department of Rheumatology and Clinical Immunology from January 2007 to June 2021. We analyzed patients’ medical data. The included patients met the CASPAR criteria for PsA diagnosis [12]. Every included patient had a PsO skin disease and joint affection (axial, peripheral, or combined). All of them were treated with either TNFα or IL-17 inhibitors. Other drugs and comorbidities, as well as body weight and height, were recorded. Body mass index (BMI) was calculated using the following formula: body mass/height^2^ (kg/m^2^). Obesity was defined as BMI ≥ 30 kg/m^2^. Patients with other co-existing autoimmune inflammatory diseases (listed in Table 1) were excluded. In our center we apply bDMARDs only to patients with normal renal function. We analyzed disease activity using the DAS28 score and patients’ pain using a visual analog scale (VAS) as a part of DAS28, and we recorded the number of swollen and tender joints, SUA levels, erythrocyte sedimentation rate (ESR), C reactive protein (CRP), and lipid profile at the start of bDMARD treatment and after 3–6 months. SUA levels were determined via photometry with uricase and peroxidase (Roche Diagnostics, Mannheim, Germany) at the Department of Laboratory Diagnostics. Hyperuricemia is defined as SUA levels that are ≥450 µmol/L (7.6 mg/dL) for males and ≥360 µmol/L (6 mg/dL) for females [3,10]. We recorded the concomitant use of glucocorticoid therapy and csDMARDs, namely, methotrexate (MTX), leflunomide (LEF), and sulfasalazine (SSZ). Patients treated with SUA-lowering agents (listed in Table 1) were not included in our study. According to the current recommendations for bDMARD efficacy, the first assessment is usually after a 3–4-month (12–16 weeks) period [13]. Sometimes, it is postponed because of objective reasons, such as an infection. Therefore, the most objective time period for evaluation is 3–6 months, and each of the above-mentioned variables was recorded at the initiation of bDMARD treatment and after a follow-up period [13]. A flowchart of included patients’ data is shown on Figure 1.

Statistical analysis was performed using the statistical software Statistica 13.0 (TIBCO, Palo Alto, CA, USA) and SPSS 27 (IBM, New York, NY, USA). Descriptive statistics were provided using the average and standard deviation (SD). A *t*-test for dependent variables was used to compare SUA levels before and during bDMARD treatment and other documented therapies. When analyzing the effects of multiple therapies on SUA levels, a *t*-test for two independent groups was used. Normality of distributions were tested by Kolmogorov–Smirnov test and numeric variables were normally distributed (SUA levels before therapy max d = 0.070: *p* > 0.05, SUA levels after 3–6 months of therapy max d = 0.121: *p* > 0.05, age max d = 0.114: *p* > 0.05, PsA duration max d = 0.130: *p* > 0.05, BMI max d = 0.098: *p* > 0.05). A χ2 test was used to assess the difference in SUA-lowering potential between TNFα and IL-17 inhibitors. Correlations between SUA levels and PsA duration, disease activity, and cholesterol and triglyceride levels were analyzed using the Pearson correlation coefficient. A *t*-test for independent variables was used for the assessment of the correlation between decreases in SUA and the presence of hypercholesterolemia or hypertriglyceridemia. The effect of body mass index class on decreases in SUA, and effects of comorbidities (arterial hypertension, type 2 diabetes, and cardiovascular comorbidities) were analyzed using an ANOVA test and the Pearson correlation coefficient. The significance threshold for *p*-values was established at 5%.

## 3. Results

The study included 87 patients. The demographic characteristics, comorbidities, and laboratory parameters are shown in Table 2. Only one-third of our patients had a normal BMI (18.5–24.9 kg/m^2^), while the others were overweight or obese. The most common comorbidities were hypercholesterolemia and arterial hypertension, followed by hypertriglyceridemia. Considering the lipid profile, the average serum cholesterol level was 5.7 ± 1.21 mmol/L, and the average triglyceride level was 2.04 ± 1.32 mmol/L; both were slightly increased [14,15]. A total of 52 (59.8%) patients were treated with TNFα inhibitors, and 35 (40.2%) were treated with IL-17 inhibitors. The most commonly used bDMARD was secukinumab (SEC), followed by adalimumab (ADA) and golimumab (GOL). The most commonly used csDMARD was MTX, and one-third of the patients used low-dose glucocorticoid therapy (≤7.5 mg of prednisolone daily). The details of the therapies are shown in Table 3.

After a 3–6-month period of bDMARD administration, 63 (72.4%) patients reported a lower number of tender joints, and 62 (71.3%) patients reported a lower number of swollen joints. The average decrease in the number of tender joints was 5.1, and the average decrease in the number of tender joints was 3.3. Two patients predominantly had axial disease, so we could not consider the number of tender or swollen joints. Regarding disease activity, 79 patients (90.8%) had a decrease in the DAS28 score, and 58 (66.7%) patients reported lower disease activity in VAS. Considering inflammatory parameters, the average value of the ESR decreased by 7.4 mm/h, and the average CRP value decreased by 7.6 mg/L.

**Figure 2:**
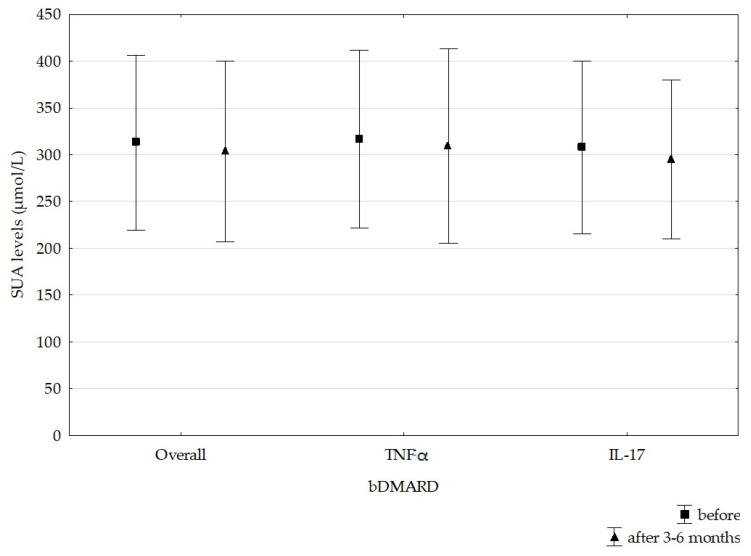
Effects of bDMARDs on SUA levels. bDMARD—biological disease-modifying antirheumatic drug, IL-17—interleukin-17, SUA—serum uric acid, TNFα—tumor necrosis factor α.

**Figure 3:**
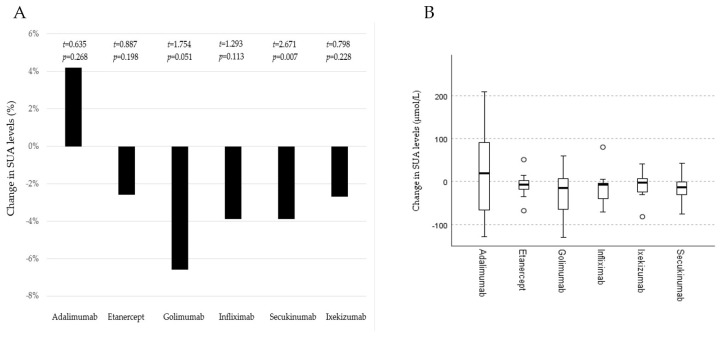
Average changes in serum uric acid (SUA) levels during the follow-up period considering therapies with different biological disease-modifying antirheumatic drugs. Part (**A**) presents percentages of average change in SUA levels. Part (**B**) presents medians, interquartile ranges, quartiles, minimums, and maximums of SUA levels changes. Certolizumab patients were not included due to the small sample size (*n* = 2). A *t*-test for two independent groups was used.

Hyperuricemia was detected in 25 (28.7%) patients—16 males and 9 females—before the initiation of bDMARDs. A significant decrease in SUA levels was noticed after 3–6 months in patients treated with bDMARDs (t = 1.773, *p* = 0.039), with an average decline of 9.4 ± 49.5 µmol/L (0.16 ± 0.83 mg/dL). In patients treated with TNF inhibitors, the average decrease in SUA was 7.25 ± 59.8 µmol/L (0.12 ± 1.01 mg/dL) (t = 0.87, *p* = 0.386), and in patients treated with IL-17 inhibitors, it was 12.6 ± 28.4 µmol/L (0.21 ± 0.48 mg/dL) (t = 2.63, *p* = 0.013). However, we did not find a statistically significant difference between these two groups (**χ**^2^ = 0.17, *p* = 0.684) (Figure 2). The most prominent average SUA decrease was noticed in patients treated with GOL (23 µmol/L), followed by infliximab (IFX) (15.8 µmol/L) and SEC (14.3 µmol/L). A decrease in SUA levels was recorded in 81.8% of patients treated with IFX, followed by 76% of those treated with SEC and 72.7% of those treated with etanercept (ETN). A statistically significant decrease in SUA levels was recorded only in the SEC group (Figure 3), but the analysis was performed on a small sample. Only in patients treated with ADA did we not document the average SUA decrease (Figure 3). Overall, 60 (69%) patients had lower SUA levels after 3–6 months of bDMARD treatment. Hyperuricemia was persistent in 21 (24.1%) patients (16 males and 5 females) after the follow-up period. 

Additional analysis was performed in patients receiving csDMARDs alongside bDMARDs. After 3–6 months of follow-up, 69.8% of the patients on MTX had lower SUA, in addition to 63.2% of the patients on LEF, 85.7% of the patients on SSZ, and 80% of the patients with two csDMARDs. There were no statistically significant effects of csDMARDs on SUA levels; for MTX, *p* = 0.489; for LEF, *p* = 0.232; for SSZ, *p* = 0.421; for two DMARDs, *p* = 0.15. Patients on low-dose glucocorticoid therapy had lower levels of SUA before (t = 2.233, *p* = 0.014) and after (t = 2.789, *p* = 0.003) 3–6 months of bDMARDs than patients without glucocorticoid therapy, but the decrease in SUA was not statistically significant (t = 1.113, *p* = 0.134). No correlations were found between the average SUA decrease and BMI (r = 0.0068, *p* = 0.48) or for SUA decrease and BMI classes (f = 0.32, *p* = 0.363). No correlations were found between average SUA decrease and comorbidities: arterial hypertension (f = 0.0043, *p* = 0.95), type 2 diabetes (f = 0.0628, *p* = 0.80), and cardiovascular comorbidities (f = 0.108, *p* = 0.92). Hypercholesterolemia and hypertriglyceridemia were not related to the levels of SUA or to the average SUA decrease considering bDMARD therapy. Patients with longer disease durations had lower average levels of SUA before (r = −0.263, *p* = 0.007) and after (r = −0.206, *p* = 0.028) 3–6 months of bDMARDs, but the disease duration was not related to the SUA decrease (r = 0.061, *p* = 0.288). Different disease activity parameters did not correlate with levels of SUA or with SUA variations (for DAS28, r = −0.22, *p* = 0.065; for VAS, r = −0.08, *p* = 0.479; for the number of swollen joints, r = 0.211, *p* = 0.055; for the number of painful joints, r = −0.147, *p* = 0.185).

## 4. Discussion

To the best of our knowledge, this is the first study on the effect of biological therapy on SUA levels in patients with PsA. Hyperuricemia is common in patients with PsA, as are hypercholesterolemia and arterial hypertension. According to a Canadian study, around one-third of patients with PsA have increased levels of SUA, which is similar to our cohort [4]. It seems that hyperuricemia could be a risk factor for the development of PsA in patients with PsO [16]. Interestingly, according to recent Italian study, hyperuricemia does not modify articular manifestations of PsA [17].

Page et al. showed that proinflammatory cytokines can activate xanthine oxidoreductase (XO) in human mammary epithelial cells, resulting in elevated SUA levels [18]. Considering the above, we expected that anti-inflammatory therapy would reduce SUA levels, as shown in the results of our research. Moreover, our results suggest that different bDMARDs have a relevant impact on levels of SUA. When we analyzed the decline in SUA levels according to the different cytokine inhibitors, we noticed the most significant decrease in patients treated with IL-17 inhibitors. However, individually, the most pronounced decrease in SUA was recorded in patients treated with GOL, a TNFα inhibitor. The results of recent studies on the effects of bDMARDs on SUA levels are contradictory. Zhao et al. showed that SEC successfully lowers SUA levels in patients with PsO, but a similar effect was not documented for the TNFα inhibitor ADA [19]. In our study, we also did not notice a decrease in SUA levels in patients treated with ADA. However, Hagino et al. showed the opposite results [20]. In our study, patients treated with GOL, IFX, and ETN had lower levels of SUA after a period of 3–6 months. According to a Czech study, TNFα inhibitors were related to an increase in SUA after the first 3 months of treatment, while CRP or IL-6 were related to a decrease [21]. However, their cohort included patients with different types of arthritis (PsA, rheumatoid arthritis, ankylosing spondylitis, and adults with juvenile idiopathic arthritis); therefore, the results cannot be compared. Still, it seems that ankylosing spondylitis patients treated with TNFα inhibitors have lower levels of SUA than patients treated only with non-steroidal anti-inflammatory drugs [22]. As we expected, bDMARDs had a positive effect on PsA activity.

Hyperuricemia is frequent in patients with PsO in Western Europe [23]. So, the severity of PsO could be a possible factor for more significant SUA decrease in patients treated with IL-17 inhibitors when compared to TNFα inhibitors. However, results from a recent observational study with more than 20,000 participants did not confirm a relationship between SUA and PsO [24]. However further analysis showed a relationship between PsO and SUA only in female patients [25]. As we mentioned above, Zhao et al. described a favorable effect of SEC on SUA in PsO patients [19].

PsA is associated with CV comorbidities, just like hyperuricemia [1,2]. In light of PsA pathogenesis, we can consider hyperuricemia as a possible risk factor for CV events [3,8,9]. More than two-thirds of our patients were overweight or obese, further increasing the risk of multiple morbidities in PsA, particularly CV and/or metabolic comorbidities [26]. PsA patients have a higher risk of CV incidents than patients with PsO but without PsA [27]. In addition, hyperuricemia is associated with increased CV risk [8,9]. So, we can speculate that lowering SUA levels with bDMARDs in patients with PsA could have a positive effect on CV and metabolic comorbidities. Probably the strongest evidence of CV risk reduction is a study with a large cohort of PsO patients from the United States treated with TNFα inhibitors [28]. Another study showed that TNFα inhibitors improved aortic stiffness in patients with inflammatory arthropathies [29]. In our cohort, SUA levels were not related to other CV risk factors, such as BMI, hypercholesterolemia, or hypertriglyceridemia, as we expected.

Concomitant csDMARD therapy did not significantly affect SUA reduction as part of bDMARD therapy. The role of csDMARDs in our study was confounded due to the small cohort and short follow-up period, which was a weakness of our study. Patients receiving low-dose prednisolone had significantly lower SUA levels before and after the follow-up period, but we did not notice a significant decrease in SUA levels in those patients. Lower SUA levels in patients receiving low-dose prednisolone are expected, since prednisolone is one of the therapeutic options in the treatment of gout, and the reduction in SUA levels is a consequence of a significant increase in renal uric acid clearance [30]. So, we deem that bDMARDs had a dominant role in the SUA decline in our cohort, in spite of csDMARD and low-dose prednisolone therapies, whose effects on the SUA decline were not statistically confirmed. In addition to that mentioned above, another limitation of our study was the heterogeneity of the included patients regarding bDMARDs, so a more detailed analysis could not be performed. Our cohort was not randomized, and the data were collected from medical documentation. Furthermore, we did not validate severity of PsO and its effect on SUA levels, as our data were collected at the rheumatologic clinic. As this was a retrospective study, a control or placebo group could not be included.

## 5. Conclusions

Our study demonstrated a positive effect of bDMARDs on SUA levels in PsA. Based on these results, we can conclude that bDMARDs lower SUA levels. Compared with TNF inhibitors, IL17 inhibitors have a more potent effect. However, further research is needed to clarify the effects of bDMARDs on SUA levels considering different aspects of psoriatic disease, such as axial or peripheral arthritis or PsO severity.

## Figures and Tables

**Table 1:** Exclusion criteria.

Diseases	Medications
Skin psoriasis without joint affection	Alopurinol
Rheumatoid arthritis	Febuxostat
Spondyloarthritis	Colchicine
Ankylosing spondylitis	Pegloticase
Reactive arthritis	Probenecid
Arthritis associated with inflammatory bowel disease	Uricase
Adults with juvenile idiopathic arthritis	
Systemic lupus erythematosus	
Sjögren’s syndrome	
Inflammatory myopathies	
Systemic sclerosis	
Systemic vasculitis	
Sarcoidosis	

**Table 2:** Demographic data.

	Total (*n* = 87)	Females (*n* = 46)	Males (*n* = 41)
Age, mean (S.D. ^1^), years	53.9 (11.5)	54.9 (12.5)	52.7 (10.3)
PsA ^2^ duration, mean (S.D.), years	10.8 (7.2)	10.9 (7.5)	10.6 (6.9)
BMI, mean (S.D.), kg/m^2^	27.7 (4.1)	27.8 (4.4)	27.5 (3.7)
Normal BMI ^3^	24 (27.6)	15 (32.6)	9 (21.9)
Overweight BMI	39 (44.8)	18 (39.1)	21 (51.2)
Obese BMI	24 (27.6)	13 (28.3)	11 (26.8)
Hypercholesterolemia	53 (60.9)	27 (58.7)	26 (63.4)
Hypertriglyceridemia	33 (37.9)	13 (28.3)	20 (48.8)
Arterial hypertension	52 (59.8)	28 (60.9)	24 (58.5)
Type 2 diabetes	16 (18.4)	8 (17.4)	8 (19.5)
Cardiovascular comorbidities	10 (11.5)	6 (13.1)	4 (9.8)
Smoking	5 (5.8)	5 (10.9)	0 (0)

Data are presented as *n* (%) unless otherwise noted. ^1^ standard deviation; ^2^ psoriatic arthritis; ^3^ body mass index.

**Table 3:** PsA therapy.

Therapy Option		Patients (*n* = 87)
TNFα inhibitors	Adalimumab	14 (16.1)
	Etanercept	11 (12.6)
	Golimumab	14 (16.1)
	Infliximab	11 (12.6)
	Certolizumab pegol	2 (2.3)
IL-17 inhibitors	Secukinumab	25 (28.7)
	Ixekizumab	10 (11.5)
csDMARD	Methotrexate	43 (49.4)
	Leflunomide	19 (21.8)
	Sulfasalazine	7 (8.1)
	Two or more csDMARDs	10 (11.5)
Low-dose glucocorticoid (≤7.5 mg prednisolone)		28 (32.2)

Data are presented as *n* (%).

## Data Availability

The data presented in this study are available from the corresponding author upon request. The data are not publicly available because some of them consist of patient information.

## References

[1] Perez-Chada L.M., Merola J.F. (2020). Comorbidities associated with psoriatic arthritis: Review and update. Clin. Immunol..

[2] Ogdie A., Schwartzman S., Husni M.E. (2015). Recognizing and managing comorbidities in psoriatic arthritis. Curr. Opin. Rheumatol..

[3] AlJohani R., Polachek A., Yang Y.J., Chandran V., Gladman D.D. (2018). Characteristic and Outcome of Psoriatic Arthritis Patients with Hyperuricemia. J. Rheumatol..

[4] Bruce I.N., Schentag C.T., Gladman D.D. (2000). Hyperuricemia in psoriatic arthritis: Prevalence and associated features. J. Clin. Rheumatol..

[5] Liu Y., Zhao Q., Yin Y., McNutt M.A., Zhang T., Cao Y. (2018). Serum levels of IL-17 are elevated in patients with acute gouty arthritis. Biochem. Biophys. Res. Commun..

[6] Raucci F., Iqbal A.J., Saviano A., Minosi P., Piccolo M., Irace C., Caso F., Scarpa R., Pieretti S., Mascolo N. (2019). IL-17A neutralizing antibody regulates monosodium urate crystal-induced gouty inflammation. Pharmacol. Res..

[7] Tripolino C., Ciaffi J., Ruscitti P., Giacomelli R., Meliconi R., Ursini F. (2021). Hyperuricemia in Psoriatic Arthritis: Epidemiology, Pathophysiology, and Clinical Implications. Front. Med..

[8] Rahimi-Sakak F., Maroofi M., Rahmani J., Bellissimo N., Hekmatdoost A. (2019). Serum uric acid and risk of cardiovascular mortality: A systematic review and dose-response meta-analysis of cohort studies of over a million participants. BMC Cardiovasc. Disord..

[9] Maloberti A., Giannattasio C., Bombelli M., Desideri G., Cicero A.F.G., Working Group on Uric Acid and Cardiovascular Risk of the Italian Society of Hypertension (SIIA) (2020). Hyperuricemia and Risk of Cardiovascular Outcomes: The Experience of the URRAH (Uric Acid Right for Heart Health) Project. High Blood Press. Cardiovasc. Prev..

[10] Gois P.H.F., Souza E.R.M. (2020). Pharmacotherapy for hyperuricaemia in hypertensive patients. Cochrane Database Syst. Rev..

[11] Ogdie A., Coates L.C., Gladman D.D. (2020). Treatment guidelines in psoriatic arthritis. Rheumatology.

[12] Taylor W., Gladman D., Helliwell P., Marchesoni A., Mease P., CASPAR Study Group (2006). Classification criteria for psoriatic arthritis: Development of new criteria from a large international study. Arthritis Rheum..

[13] Simons N., Degboé Y., Barnetche T., Cantagrel A., Ruyssen-Witrand A., Constantin A. (2020). Biological DMARD efficacy in psoriatic arthritis: A systematic literature review and meta-analysis on articular, enthesitis, dactylitis, skin and functional outcomes. Clin. Exp. Rheumatol..

[14] Civeira F., Arca M., Cenarro A., Hegele R.A. (2022). A mechanism-based operational definition and classification of hypercholesterolemia. J. Clin. Lipidol..

[15] Pejic R.N., Lee D.T. (2006). Hypertriglyceridemia. J. Am. Board Fam. Med..

[16] Tsuruta N., Imafuku S., Narisawa Y. (2017). Hyperuricemia is an independent risk factor for psoriatic arthritis in psoriatic patients. J. Dermatol..

[17] Galozzi P., Oliviero F., Scanu A., Lorenzin M., Ortolan A., Favero M., Doria A., Ramonda R. (2022). Acute joint swelling in psoriatic arthritis: Flare or “psout”-A 10-year-monocentric study on synovial fluid. Exp. Biol. Med..

[18] Page S., Powell D., Benboubetra M., Stevens C.R., Blake D.R., Selase F., Wolstenholme A.J., Harrison R. (1998). Xanthine oxidoreductase in human mammary epithelial cells: Activation in response to inflammatory cytokines. Biochim. Biophys. Acta.

[19] Zhao Z., Cai L., Zhang S., Zhang H., Liu X., Li C., Zhao Y., Zhang J. (2022). Effects of secukinumab and adalimumab on serum uric acid level in patients with plaque psoriasis. Chin. Med. J..

[20] Hagino T., Saeki H., Fujimoto E., Kanda N. (2023). Effects of Biologic Therapy on Laboratory Indicators of Cardiometabolic Diseases in Patients with Psoriasis. J. Clin. Med..

[21] Hasikova L., Pavlikova M., Hulejova H., Kozlik P., Kalikova K., Mahajan A., Herrmann M., Stiburkova B., Zavada J. (2019). Serum uric acid increases in patients with systemic autoimmune rheumatic diseases after 3 months of treatment with TNF inhibitors. Rheumatol. Int..

[22] Sargın B., Gürer G., Taşcı Bozbaş G., Öztürk H. (2019). Association between serum uric acid and inflammation markers in ankylosing spondylitis patients treated with tumor necrosis factor-α or nonsteroidal anti-inflammatory drugs. Eur. Res. J..

[23] Li X., Miao X., Wang H., Wang Y., Li F., Yang Q., Cui R., Li B. (2016). Association of Serum Uric Acid Levels in Psoriasis: A Systematic Review and Meta-Analysis. Medicine.

[24] Hu M., Wang Y., Xu W., Bai J., Tang X. (2024). The impact of serum uric acid on psoriasis: NHANES 2005-2014 and Mendelian randomization. Front. Genet..

[25] Zhao D., Zhao J.R., Wang S., Sun J.H. (2024). Evaluating causal influence of serum uric acid on psoriasis via observational study and transethnic Mendelian randomization analyses. Sci. Rep..

[26] Kumthekar A., Ogdie A. (2020). Obesity and Psoriatic Arthritis: A Narrative Review. Rheumatol. Ther..

[27] Jamnitski A., Symmons D., Peters M.J., Sattar N., McInnes I., Nurmohamed M.T. (2013). Cardiovascular comorbidities in patients with psoriatic arthritis: A systematic review. Ann. Rheum. Dis..

[28] Wu J.J., Sundaram M., Cloutier M., Gauthier-Loiselle M., Guérin A., Singh R., Ganguli A. (2018). The risk of cardiovascular events in psoriasis patients treated with tumor necrosis factor-α inhibitors versus phototherapy: An observational cohort study. J. Am. Acad. Dermatol..

[29] Angel K., Provan S.A., Fagerhol M.K., Mowinckel P., Kvien T.K., Atar D. (2012). Effect of 1-year anti-TNF-α therapy on aortic stiffness, carotid atherosclerosis, and calprotectin in inflammatory arthropathies: A controlled study. Am. J. Hypertens..

[30] Liu C., Zhen Y., Zhao Q., Zhai J.L., Liu K., Zhang J.X. (2016). Prednisone lowers serum uric acid levels in patients with decompensated heart failure by increasing renal uric acid clearance. Can. J. Physiol. Pharmacol..

